# Editorial: Application of computational tools to health and environmental sciences, Volume II

**DOI:** 10.3389/fphar.2022.1102431

**Published:** 2022-12-01

**Authors:** Patricia Ruiz, George Loizou

**Affiliations:** ^1^ Office of Innovation and Analytics, Agency for Toxic Substances and Disease Registry, Atlanta, GA, United States; ^2^ Health and Safety Executive, Buxton, United Kingdom

**Keywords:** PBPK, IVIVE, QSAR (qualitative structure-activity relationships), new approach methodologies (NAMs), computational toxicology, adverse outcome pathways

The scarcity of human data, the limitation of time and resources for experimental testing, and societal pressure to develop animal-free testing strategies are driving the field of toxicology toward new methods to generate and interpret data for chemical risk assessment (Luechtefeld and Hartung, 2017). Computational toxicology is an emerging multi-disciplinary research field that incorporates diverse disciplines such as computational sciences, bioinformatics, machine learning, chemo-informatics, systems biology, toxicogenomics, and toxicokinetics (Baskin, 2018; Ciallella and Zhu, 2019).

The ability to rapidly screen chemicals for biological activity and reduce the use of animals in toxicity testing has been the goal of regulatory toxicology for several decades. With current technological and scientific advances, scientists seek to apply high throughput screening assays and computational toxicology techniques to evaluate and screen large numbers of chemicals and drug data sets for biological activity (Ciallella and Zhu, 2019; Chang et al., 2022; Tetko et al., 2022).

The availability of *in vitro* toxicokinetic data is essential to meet the growing regulatory need to improve chemical safety assessments (Punt et al., 2020; Najjar et al., 2022). With the availability of a growing number of commercial and open-source computational tools and databases for quantitative structure-activity relationship (QSAR) and physiologically-based pharmacokinetic (PBPK) modeling, scientists are developing workflows that integrate new approach methodologies (NAMs), *in silico* techniques and *in vitro* data to predict biological activity in humans (Loizou et al., 2008; Ruiz et al., 2020).

This research topic on applying computational tools to health and environmental sciences brings together exemplars of current modeling efforts that seek to play an important role in next-generation risk assessment, such as:

• Understanding toxicokinetics of xenobiotics based on omics approaches
• Development and acceptance of high-throughput computational tools
• Building data-driven models to identify chemicals associated with adverse outcome pathways (AOPs) and toxicity
• Development and application of a computational workflow for probabilistic quantitative *in vitro* to *in vivo* extrapolation (QIVIVE) in environmental risk assessment
• Data harmonization and curation from human studies

The overall aim is to develop and engage model developers and users to promote and maximize benefits from basic and applied research that is being undertaken to protect public and environmental health.

Nicolas et al. presented a case study demonstrating the utility of exploiting existing computational methods (ratio of surrogate hazard and exposure data, called margins of exposure) at the pre-assessment phase of a tiered risk-based approach to prioritize thousands of untested chemicals quickly and conservatively for further research.

Pierro et al. developed a data-driven model to identify chemicals associated with all-trans retinoic acid (ATRA) pathway bioactivity and prenatal skeletal defects. The model provides potential avenues for new mechanistic discoveries related to ATRA pathway disruption and associated skeletal dysmorphogenesis due to environmental exposures.

Khalidi et al. presented an R-based workflow for automated high-throughput PBK simulation with the Simcyp ^®^ simulator called “SimRFlow.” It is a time-efficient tool for simulating the biokinetics of many compounds without the manual curation of physicochemical or experimental data necessary to run Simcyp^®^ simulations.

Mandal et al. performed a correlation analysis of variables from the Atherosclerosis Risk in Communities (ARIC) Study. The developed workflow could be incorporated into data harmonization efforts to reduce the human effort required for initial variable mapping and provide crucial quantitative information to assist with the harmonization.

Zhao et al. provided a basis for exploring toxicokinetics, toxicity, and valuable mechanistic insight. Integrating toxicokinetics of arenobufagin (ArBu) with lipidomics and proteomics approaches based on liquid chromatography-tandem mass spectrometry (LC-MS/MS) can accurately identify and quantitatively compare proteins, which is of great significance in the determination of ArBu cardiotoxicity and efficacy mechanisms.

Tan et al. developed a physiologically-based pharmacokinetic (PBPK) model capable of simulating cefadroxil concentrations in plasma and tissues in mice, rats, and humans. The model is helpful for dose selection and informative decision-making during clinical trials and dosage form design of cefadroxil and provides a reference for the PBK studies of hPEPT1 substrate.

Loizou et al. provided a state-of-the-art computational workflow that integrates PBK modeling, global sensitivity analysis (GSA), approximate Bayesian computation (ABC), Markov Chain Monte Carlo (MCMC) simulation, and the Virtual Cell Based Assay (VCBA) for the estimation of the active, free *in vitro* concentration of a chemical in the reaction medium were developed to facilitate quantitative *in vitro* to *in vivo* extrapolation (QIVIVE).

In summary, this research topic presents some recent developments and perspectives on the challenges in computational toxicological modeling, their applications, integration of data and tools, and their potential to meet different case scenarios within environmental and human health risk assessment. The development of computational toxicology tools and approaches should accelerate with the availability of open-source toxicological databases to provide a more quantitative, biologically based assessment of the risk of chemicals in the environment.
